# Identification of key long non-coding RNA-associated competing endogenous RNA axes in Brodmann Area 10 brain region of schizophrenia patients

**DOI:** 10.3389/fpsyt.2022.1010977

**Published:** 2022-11-03

**Authors:** Hani Sabaie, Mahdi Gholipour, Mohammad Reza Asadi, Samin Abed, Mirmohsen Sharifi-Bonab, Mohammad Taheri, Bashdar Mahmud Hussen, Serge Brand, Seyedeh Morvarid Neishabouri, Maryam Rezazadeh

**Affiliations:** ^1^Clinical Research Development Unit of Tabriz Valiasr Hospital, Tabriz University of Medical Sciences, Tabriz, Iran; ^2^Department of Medical Genetics, School of Medicine, Shahid Beheshti University of Medical Sciences, Tehran, Iran; ^3^Department of Medical Genetics, Faculty of Medicine, Tabriz University of Medical Sciences, Tabriz, Iran; ^4^Urology and Nephrology Research Center, Shahid Beheshti University of Medical Sciences, Tehran, Iran; ^5^Institute of Human Genetics, Jena University Hospital, Jena, Germany; ^6^Department of Pharmacognosy, College of Pharmacy, Hawler Medical University, Erbil, Iraq; ^7^Center of Research and Strategic Studies, Lebanese French University, Erbil, Iraq; ^8^Center for Affective, Stress and Sleep Disorders, Psychiatric Clinics of the University of Basel, Basel, Switzerland; ^9^Department of Psychiatric, School of Medicine, Shahid Beheshti University of Medical Sciences, Tehran, Iran

**Keywords:** bioinformatics analysis, competing endogenous RNA, long non-coding RNA, microarray, schizophrenia

## Abstract

Schizophrenia (SCZ) is a serious mental condition with an unknown cause. According to the reports, Brodmann Area 10 (BA10) is linked to the pathology and cortical dysfunction of SCZ, which demonstrates a number of replicated findings related to research on SCZ and the dysfunction in tasks requiring cognitive control in particular. Genetics' role in the pathophysiology of SCZ is still unclear. Therefore, it may be helpful to understand the effects of these changes on the onset and progression of SCZ to find novel mechanisms involved in the regulation of gene transcription. In order to determine the molecular regulatory mechanisms affecting the SCZ, the long non-coding RNA (lncRNA)-associated competing endogenous RNAs (ceRNAs) axes in the BA10 area were determined using a bioinformatics approach in the present work. A microarray dataset (GSE17612) consisted of brain post-mortem tissues of the BA10 area from SCZ patients and matched healthy subjects was downloaded from the Gene Expression Omnibus (GEO) database. This dataset included probes for both lncRNAs and mRNAs. Using the R software's limma package, the differentially expressed lncRNAs (DElncRNAs) and mRNAs (DEmRNAs) were found. The RNA interactions were also discovered using the DIANA-LncBase and miRTarBase databases. In the ceRNA network, positive correlations between DEmRNAs and DElncRNAs were evaluated using the Pearson correlation coefficient. Finally, lncRNA-associated ceRNA axes were built by using the co-expression and DElncRNA-miRNA-DEmRNA connections. We identified the DElncRNA-miRNA-DEmRNA axes, which included two key lncRNAs (*PEG3-AS1, MIR570HG*), seven key miRNAs (*hsa-miR-124-3p*, hsa-miR-17-5p, hsa-miR-181a-5p, *hsa-miR-191-5p, hsa-miR-26a-5p, hsa-miR-29a-3p, hsa-miR-29b-3p*), and eight key mRNAs (*EGR1, ETV1, DUSP6, PLOD2, CD93, SERPINB9, ANGPTL4, TGFB2*). Furthermore, DEmRNAs were found to be enriched in the “AGE-RAGE signaling pathway in diabetic complications”, “Amoebiasis”, “Transcriptional misregulation in cancer”, “Human T-cell leukemia virus 1 infection”, and “MAPK signaling pathway”. This study offers research targets for examining significant molecular pathways connected to the pathogenesis of SCZ, even though the function of these ceRNA axes still needs to be investigated.

## Introduction

Schizophrenia (SCZ) is a mentally destructive ailment affecting young adults. Its symptoms comprise a spectrum of delusions, hallucinations, and cognitive impairment (1). About one percent of the population is affected by this condition on a worldwide scale (2). It is believed that complicated interactions between genetic and environmental factors contribute to the pathophysiology of SCZ (3, 4). Transcriptional changes in the post-mortem human brain that are correlated with SCZ have been appraised in various studies using microarray or RNA-sequencing (5). Such studies revealed alterations in the expression of genes involved in a variety of biological processes in various parts of the brain, including the anterior prefrontal cortex (Brodmann area 10; BA10) (6–8). To devise new diagnostic techniques and individualized therapies for SCZ, we need to augment our knowledge of molecular processes that are involved in the onset and exacerbation of SCZ. Recent findings about novel mechanisms of gene expression regulation can be useful in understanding how changes in these regulatory mechanisms can affect SCZ formation and progression (9). Non-coding RNAs (ncRNAs) play an important role in the regulation of gene expression. One of the proposed mechanisms for the involvement of these transcripts in the control of gene expression is the competing endogenous RNA (ceRNA) hypothesis (10). In this novel regulatory mechanism, ncRNAs, especially long non-coding RNAs (lncRNAs), regulate other RNA transcripts through sponging shared microRNAs (miRNAs) (11). Cross-talk between RNAs, including coding RNAs and ncRNAs, through miRNA complementary sequences known as miRNA response elements (MREs), produces a large-scale regulatory network throughout the transcriptome, according to this notion. If two RNA transcripts regulate each other by a ceRNA-mediated mechanism, then the expression levels of these two RNA transcripts would be negatively correlated with the levels of target miRNAs and positively correlated with each other (11).

Although multiple investigations have shown that ceRNA networks differ in SCZ (9, 12–14), the role of ceRNA networks in the pathogenesis of SCZ remains to be elucidated, and it is of great importance to evaluate the expression of downstream affected genes by these networks. With the emergence of high throughput techniques such as RNA-sequencing and microarray, assessing the expression of numerous genes simultaneously became possible, and a bulk amount of information about the expression profile of different samples has been made publicly available for the scientific community. Different computational methods can be utilized to analyze these bulk data to unravel potential regulatory mechanisms like ncRNA networks that contribute to SCZ (9, 13, 14). RNAs are more “druggable” than proteins because they can be simply targeted by corresponding complementary sequences. These unique features make it easier and economical to design and develop drugs targeting RNA molecules as novel therapeutic targets (15–17). CeRNA networks contain a variety of transcripts, which makes them useful for exploring possible treatment targets for complex diseases like SCZ, even if only one of them is targeted (i.e., an immediate fluctuation in the levels of various disease-related RNAs) (18).

The objective of this bioinformatics-based study was to identify the expression patterns and relevant lncRNA-associated ceRNA regulatory axes in the BA10 brain region of SCZ patients.

## Methods

In the current study, a microarray dataset (GSE17612) consisting of brain post-mortem tissues of the BA10 area from SCZ patients and matched healthy subjects was analyzed. Differentially expressed lncRNAs (DElncRNAs) and mRNAs (DEmRNAs) were extracted from the dataset, and lncRNA-miRNA-mRNAs axes were constructed by utilizing previously published bioinformatic approaches (13).

### Data collection of the gene expression profile

We obtained the GSE17612 dataset from the NCBI Gene Expression Omnibus database (GEO, https://www.ncbi.nlm.nih.gov/geo/). In this dataset, the GPL570 platform (HG-U133_Plus_2) was used as microarray chip that included probes for mRNAs and lncRNAs. The GSE17612 dataset contains brain post-mortem tissues of the BA10 area from 28 patients with SCZ and 23 matched healthy subjects (8). Demographic information is shown in Table 1. According to the original study, RNA integrity number (RIN) was employed as the major inclusion criterion to assess the quality of the RNA. The RIN was evaluated using spectrophotometry to estimate the amount of extracted RNA, and an Agilent 2100 Bioanalyzer (South Plainfield, NJ, USA) was used to evaluate the RNA's quality. The RIN was used to classify samples into three quality categories: pass (RIN > 7.0), borderline (RIN 6.0-7.0), and fail (RIN < 6.0) (8). Five samples in the fail group were excluded from the study after classification. In addition, six samples were excluded following the evaluation of hybridization quality (8). A total of 51 samples were included (8).

**Table 1:** Demographic information.

**Sample**	**Source name**	**Age**	**Gender**	**Ph**	**Post-mortem delay (h)**
GSM439778	Brain BA10 post-mortem schizophrenic	74	Male	6	4.5
GSM439779	Brain BA10 post-mortem control	72	Male	6.8	12
GSM439780	Brain BA10 post-mortem control	89	Female	6.5	5
GSM439781	Brain BA10 post-mortem schizophrenic	85	Female	5.9	3.5
GSM439782	Brain BA10 post-mortem control	61	Male	6.2	16.5
GSM439783	Brain BA10 post-mortem control	54	Female	6.5	4
GSM439784	Brain BA10 post-mortem schizophrenic	41	Male	6.3	8
GSM439785	Brain BA10 post-mortem schizophrenic	71	Male	6	6.5
GSM439786	Brain BA10 post-mortem schizophrenic	77	Male	6.1	3
GSM439787	Brain BA10 post-mortem control	90	Female	5.7	12.5
GSM439788	Brain BA10 post-mortem control	91	NA	6.3	4.5
GSM439789	Brain BA10 post-mortem control	54	Male	6.6	12
GSM439790	Brain BA10 post-mortem schizophrenic	65	Female	6.1	3
GSM439791	Brain BA10 post-mortem schizophrenic	75	Male	6.3	9
GSM439792	Brain BA10 post-mortem control	78	Female	6.7	8
GSM439793	Brain BA10 post-mortem control	90	Male	6.2	6.75
GSM439794	Brain BA10 post-mortem schizophrenic	56	Male	6.4	16.5
GSM439795	Brain BA10 post-mortem schizophrenic	81	Female	NA	22
GSM439796	Brain BA10 post-mortem schizophrenic	82	Male	6	11
GSM439797	Brain BA10 post-mortem control	91	Male	6.3	9.5
GSM439798	Brain BA10 post-mortem schizophrenic	82	Male	6.4	11
GSM439799	Brain BA10 post-mortem control	58	Male	6.5	15
GSM439800	Brain BA10 post-mortem schizophrenic	28	Female	6.3	11
GSM439801	Brain BA10 post-mortem schizophrenic	87	Male	6	3.5
GSM439802	Brain BA10 post-mortem control	87	Female	6.5	14.5
GSM439803	Brain BA10 post-mortem schizophrenic	72	Male	6.6	20
GSM439804	Brain BA10 post-mortem control	25	Male	6.9	17
GSM439805	Brain BA10 post-mortem schizophrenic	82	Female	5.9	8.5
GSM439806	Brain BA10 post-mortem schizophrenic	79	Male	6.1	4.5
GSM439807	Brain BA10 post-mortem schizophrenic	88	Female	5.7	7
GSM439808	Brain BA10 post-mortem control	94	Female	6.3	9.5
GSM439809	Brain BA10 post-mortem schizophrenic	63	Male	6.5	30
GSM439810	Brain BA10 post-mortem control	46	Female	6.6	4
GSM439811	Brain BA10 post-mortem schizophrenic	75	Female	6	3
GSM439812	Brain BA10 post-mortem control	68	Female	6.4	6
GSM439813	Brain BA10 post-mortem schizophrenic	83	Male	6.1	11.5
GSM439814	Brain BA10 post-mortem control	60	Male	6.9	16
GSM439815	Brain BA10 post-mortem schizophrenic	79	Male	6.3	4.5
GSM439816	Brain BA10 post-mortem control	38	Male	6.9	6
GSM439817	Brain BA10 post-mortem schizophrenic	79	Male	6.2	4
GSM439818	Brain BA10 post-mortem control	71	Female	6.3	13
GSM439819	Brain BA10 post-mortem control	91	Male	6.3	13
GSM439820	Brain BA10 post-mortem schizophrenic	97	Female	6.2	3.5
GSM439821	Brain BA10 post-mortem schizophrenic	82	Male	5.8	19.5
GSM439822	Brain BA10 post-mortem control	25	Male	6.6	12
GSM439823	Brain BA10 post-mortem schizophrenic	82	Male	6.2	5
GSM439824	Brain BA10 post-mortem schizophrenic	44	Male	6.3	4
GSM439825	Brain BA10 post-mortem control	88	Female	6.8	5
GSM439826	Brain BA10 post-mortem control	67	Female	6.7	6
GSM439827	Brain BA10 post-mortem schizophrenic	67	Female	6.1	3.5
GSM439828	Brain BA10 post-mortem schizophrenic	77	Male	6.2	3

### Data preprocessing and identification of DEmRNAs and DElncRNAs

We employed Robust Multichip Average (RMA) method for background correction and quantile normalization of all primary data records (19). In addition, to exclude insignificant probe sets with no expression, we performed an interquartile range (IQR) filter (IQR across the samples on the log2 scale larger than median IQR), that was followed by an intensity filter (a minimum of >100 expression signals in a minimum of 25% of the arrays) (20). AgiMicroRna Bioconductor package (version 2.46.0) was used for quality control. We applied removeBatchEffect() function from the limma package and added age and gender as covariates in all statistical models to adjust for possible effects. We applied linear models for microarray data (limma) package (version 3.52.2) of R (version 4.2.1) for differential gene expression analysis between patients with SCZ and control subjects (21) in Bioconductor (https://www.bioconductor.org/) (22). We used the same approach in our previous paper to recognize lncRNA probes (13). We downloaded the latest list of lncRNAs from the HUGO Gene Nomenclature Committee (HGNC) (https://www.genenames.org/) with approved symbols (23). Then after, we merged obtained lncRNAs list with the dataset based on their gene symbols to retrieve common lncRNAs between the lncRNAs list and dataset. Student *t*-test was applied to evaluate the statistical significance of differential expression, and *P*-value adjustment was carried out using Benjamini-Hochberg method. Cut-off values applied in this study were set as follows: (1) a false discovery rate (adjusted *P*-value) < 0.001, and (2) |log2 fold change (log2FC) | > 0.5. Finally, a volcano plot and heat map of DEGs was drawn using the Enhanced Volcano (version1.14.0) and the Pheatmap (version 1.0.12) packages.

### RNA interaction pairs prediction

We utilized DIANA-LncBase v3 to identify miRNAs targeting DElncRNAs based on experimentally validated interaction (24). The DIANA-LncBase query was performed based on the “Species” of Homo Sapiens and high “miRNA Confidence Levels”. Also, we recognized miRNAs targeting mRNAs using miRTarBase (25); only the interactions with strong experimental evidence were included in the study. By comparing the mRNAs obtained from miRTarBase and the previously identified DEmRNA, the common mRNAs were used to construct the DElncRNA-miRNA-DEmRNA regulatory axes.

### Analysis of correlation between DEmRNAs and DElncRNAs, protein–protein interaction (PPI) network analysis, and lncRNA-associated ceRNA axes construction

We investigated the positive correlation between DElncRNAs and DEmRNAs in ceRNA networks using Pearson correlation analysis. DELncRNAs, targeted DEmRNAs, and the interacting miRNAs were omitted upon observing opposing expression patterns between the targeted DEmRNAs and DElncRNAs. The correlations were calculated and illustrated using bcdstats (version 0.0.0.9005) and corrplot (version 0.92) R packages. Inclusion criteria was based on Pearson correlation coefficient (*r*) > 0.5 and false discovery rate (FDR) < 0.001. The online STRING database (https://string-db.org/) (26) was utilized to create a PPI to prognosticate the interactions among DEmRNAs encoding proteins. For PPI network construction, a combined score of 0.4 (medium confidence) was selected. Visualization of these PPI and construction of ceRNA networks was carried out using Cytoscape software (version 3.8.0) (27).

### DEmRNAs pathway enrichment analysis

We performed a KEGG pathway enrichment analysis of DEmRNAs in the ceRNA network using the Enricher tool (28, 29).

## Results

### Identification of DEmRNAs and DElncRNAs

Before implementing differential expression analysis, background correction, normalization, and gene filtering were carried out. The quality control of the dataset was conducted using the AgiMicroRna Bioconductor package. To assess the distribution of data, we drew box plots of the gene expression data after performing normalization (Supplementary File 1). The medians of expression level were equal for each of the distinct arrays in the box plot, proving that the correction was done correctly.

**Figure 1:**
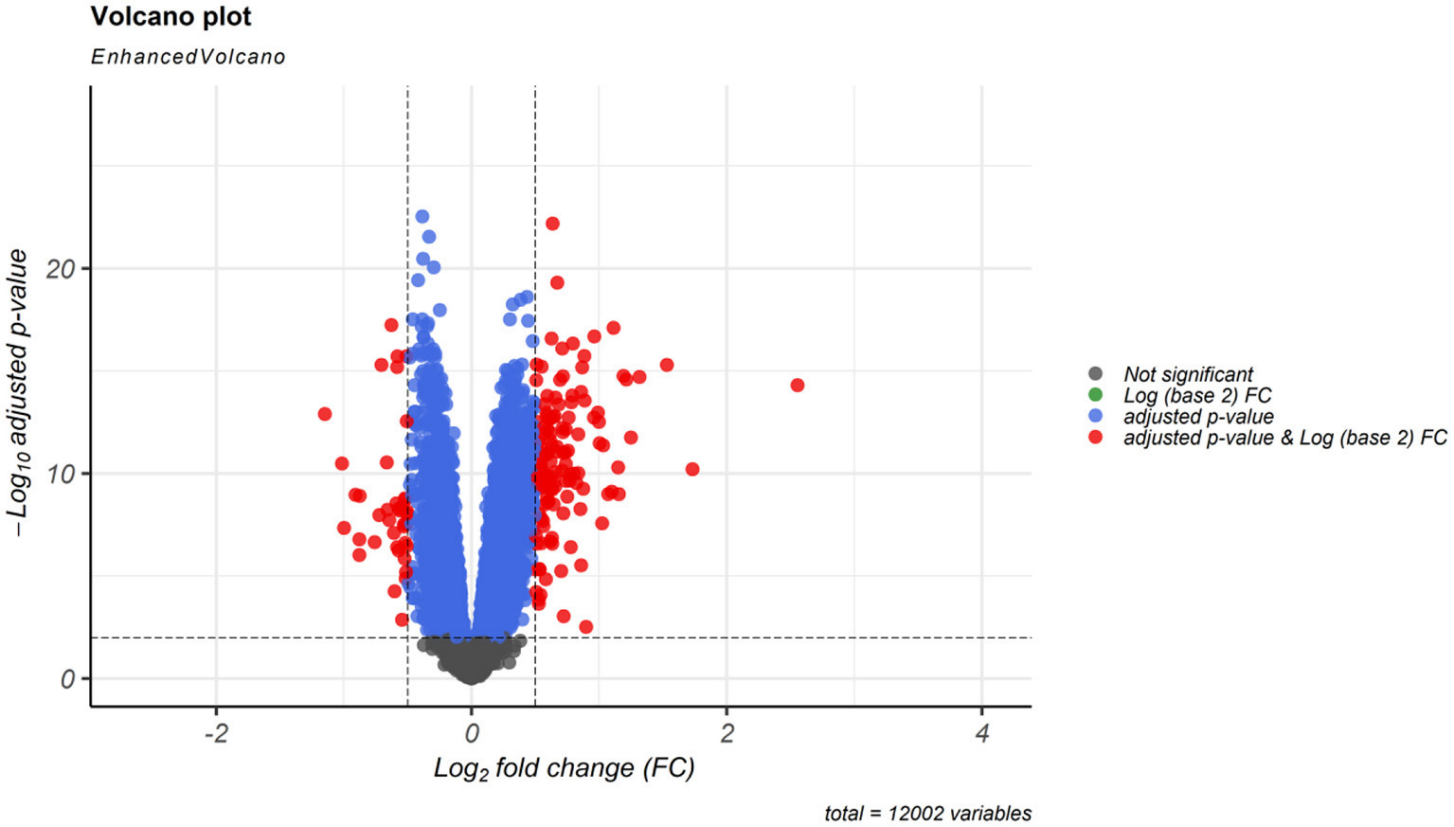
Differentially expressed mRNAs (DEmRNAs) volcano plot. DEmRNAs were screened using a |(log2FC)| ≥ 0.5 and an adjusted *P* < 0.001 threshold.

**Figure 2:**
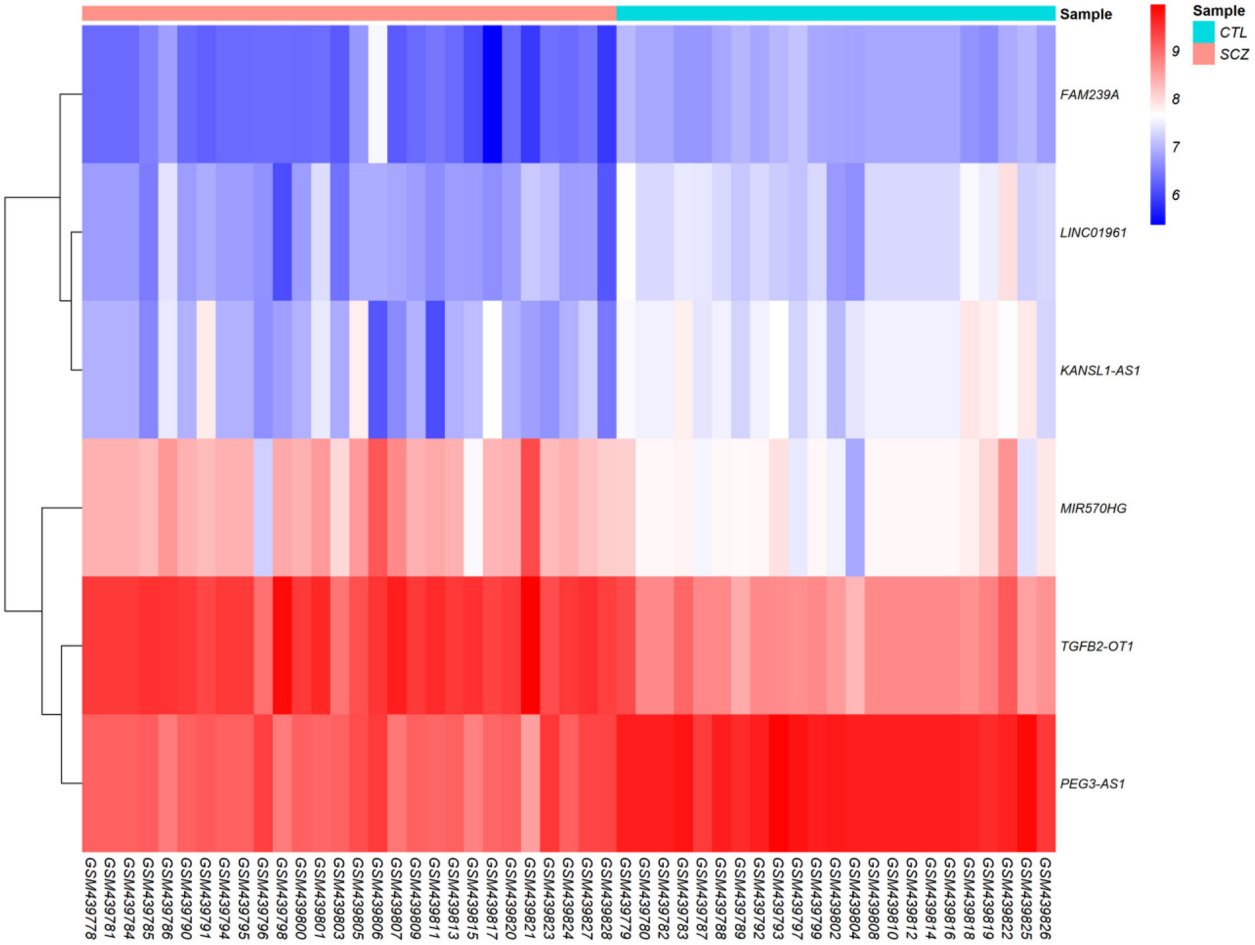
Differentially expressed long non-coding RNAs (DElncRNAs) heatmap. The color values correspond to normalized gene expression data. The color and intensity of the boxes are used to reflect the expression level of DElncRNAs in each sample. The color blue shows those DElncRNAs with low expression, while red represents those with high expression.

According to cut-off values for identification of DEGs and DElncRNAs (|log2FC | ≥ 0.5, and adjusted *P* < 0.001), a total of 183 DEmRNAs and six DElncRNAs were observed between SCZ and control samples. Figures 1, 2 represent the volcano plot of the DEmRNAs and heatmap of DElncRNAs, respectively. Also, we summarized more details about DEGs in Supplementary File 2.

### Prediction of RNA interaction pairs

We used the DIANA-LncBase ver. 3 online tool to predict miRNAs targeting DElncRNA. Results showed that the miRNAs might target three of the six DElncRNAs. Then we obtained mRNAs targeted by candidate miRNAs using miRTarBase. Lastly, we retrieved 20 overlapping genes by comparing mRNAs obtained from miRTarBase and DEmRNAs. Additional information regarding RNA interaction pairs is provided in Supplementary File 3.

### Analysis of correlation between DEmRNAs and DElncRNAs, PPI network analysis, and lncRNA-associated ceRNA axes construction

**Figure 3:**
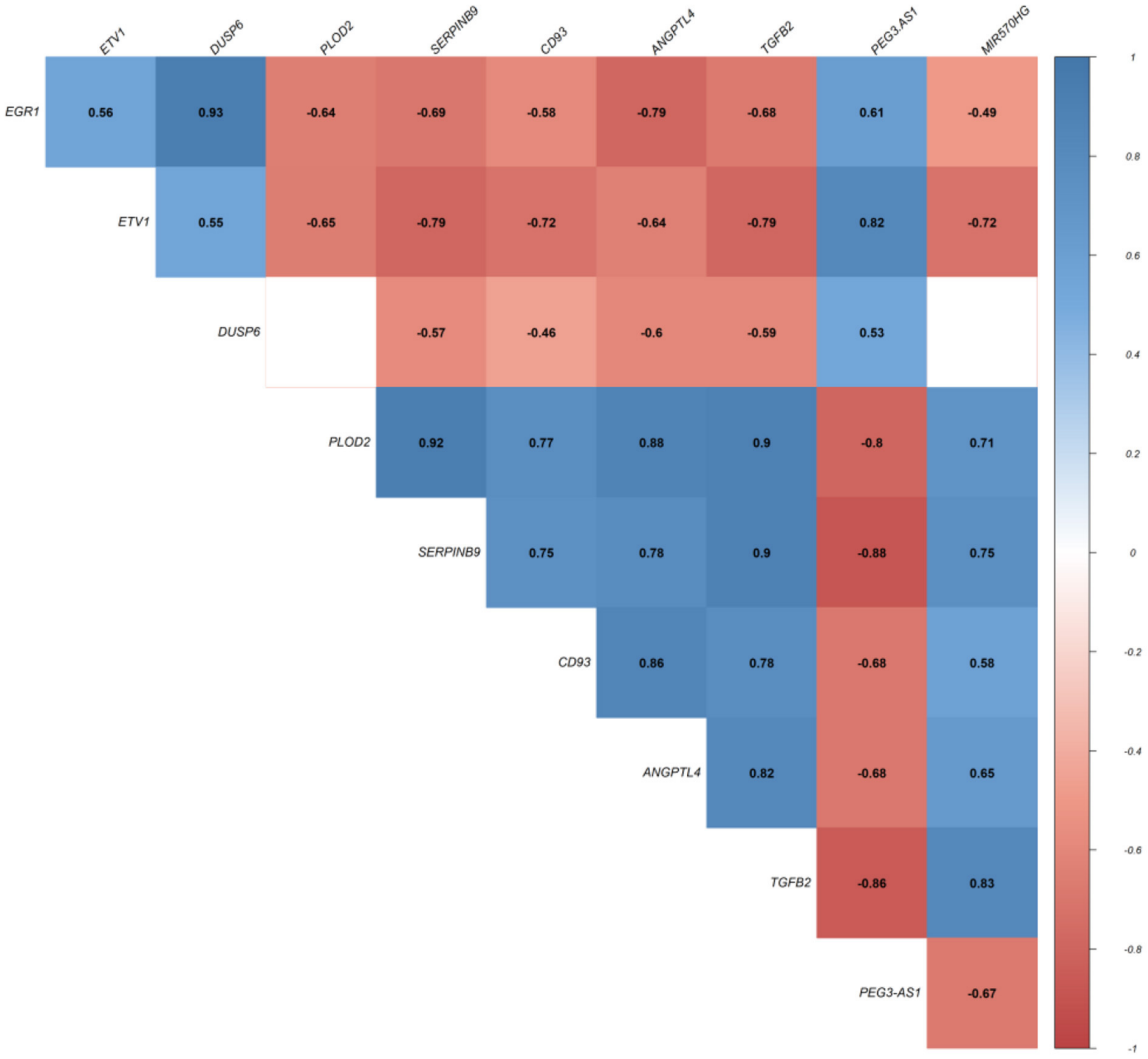
Pearson correlation analysis. Values represent Pearson's correlation coefficient (*r*). Positive correlations are shown in blue, while negative correlations are represented in red. False discovery rate (FDR) values greater than 0.001 are deemed unimportant (blank), and the intensity is proportional to correlation coefficients.

**Figure 4:**
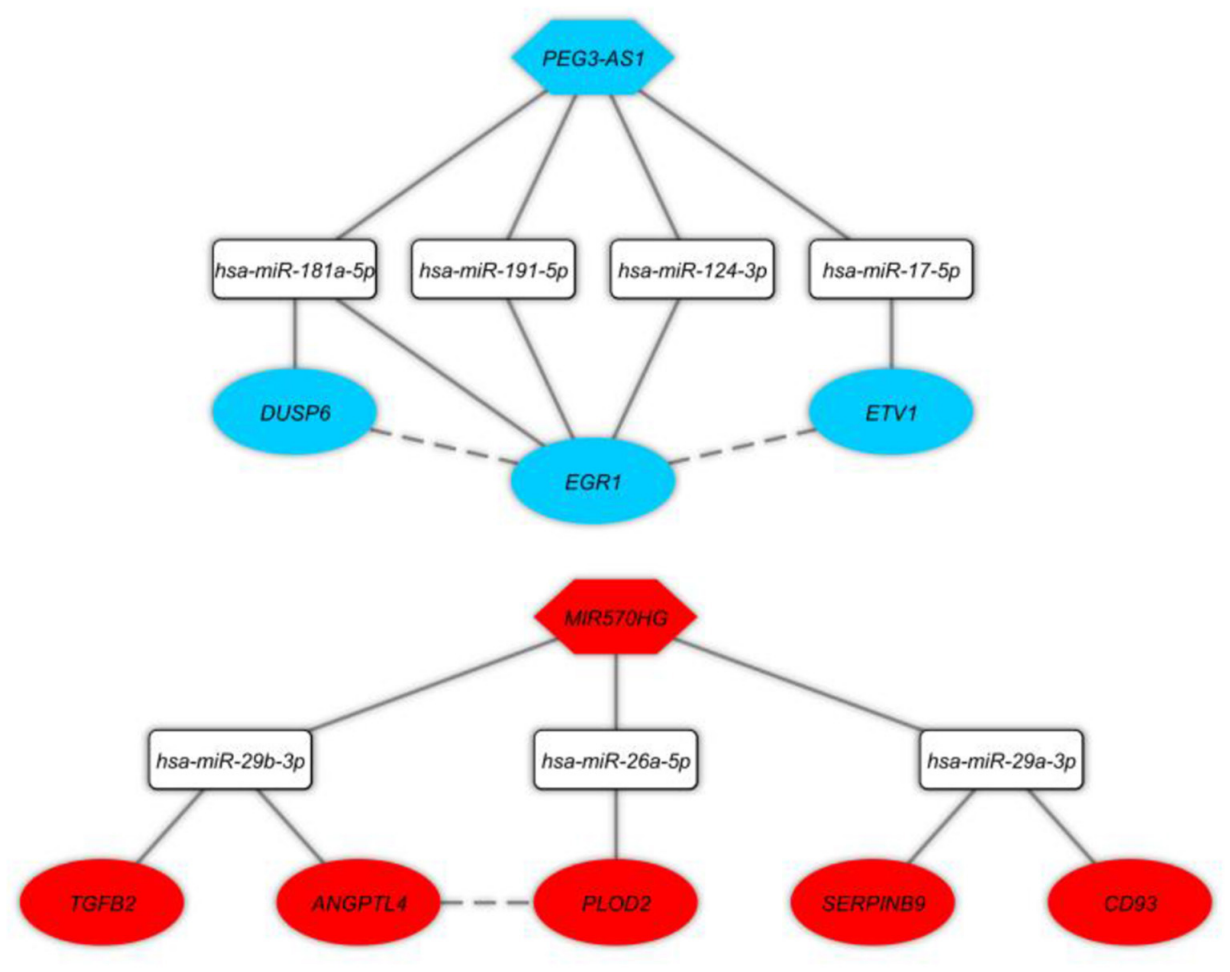
Long non-coding RNA (lncRNA)-associated competing endogenous RNA axes. Red and blue represents upregulation and downregulation of ceRNAs, respectively. LncRNAs, miRNAs, and mRNAs are represented by hexagon, round rectangle, and ellipse, respectively. The dash lines between nodes represent the interactions between proteins.

In this step, we applied Pearson correlation analysis of expression levels between DEmRNAs and DElncRNAs to explore positive correlations to establish the ceRNA hypothesis (mRNAs' expression is positively modulated by lncRNAs through sponging miRNAs; Figure 3). We constructed a ceRNA network based on interactions of DElncRNA, miRNA, and DEmRNA, co-expression pattern of DElncRNA and DEmRNA, and PPIs to indicate the lncRNAs associated ceRNA axes in the brain BA10 region that are related to SCZ (Figure 4). CeRNA axes consisted of two DElncRNAs [PEG3 Antisense RNA 1 (*PEG3-AS1*), MIR570 Host Gene (*MIR570HG*)], eight DEmRNAs [Early Growth Response 1 (*EGR1*), ETS Variant Transcription Factor 1 (*ETV1*), Dual specificity phosphatase 6 (*DUSP6*), Procollagen-Lysine, 2-Oxoglutarate 5-Dioxygenase 2 (*PLOD2*), *CD93*, Serpin Family B Member 9 (*SERPINB9*), Angiopoietin Like 4 (*ANGPTL4*), Transforming Growth Factor Beta 2 (*TGFB2*)] and seven miRNAs (*hsa-miR-124-3p, hsa-miR-17-5p, hsa-miR-181a-5p, hsa-miR-191-5p, hsa-miR-26a-5p, hsa-miR-29a-3p, hsa-miR-29b-3p*).

### DEmRNAs pathway enrichment analysis

**Figure 5:**
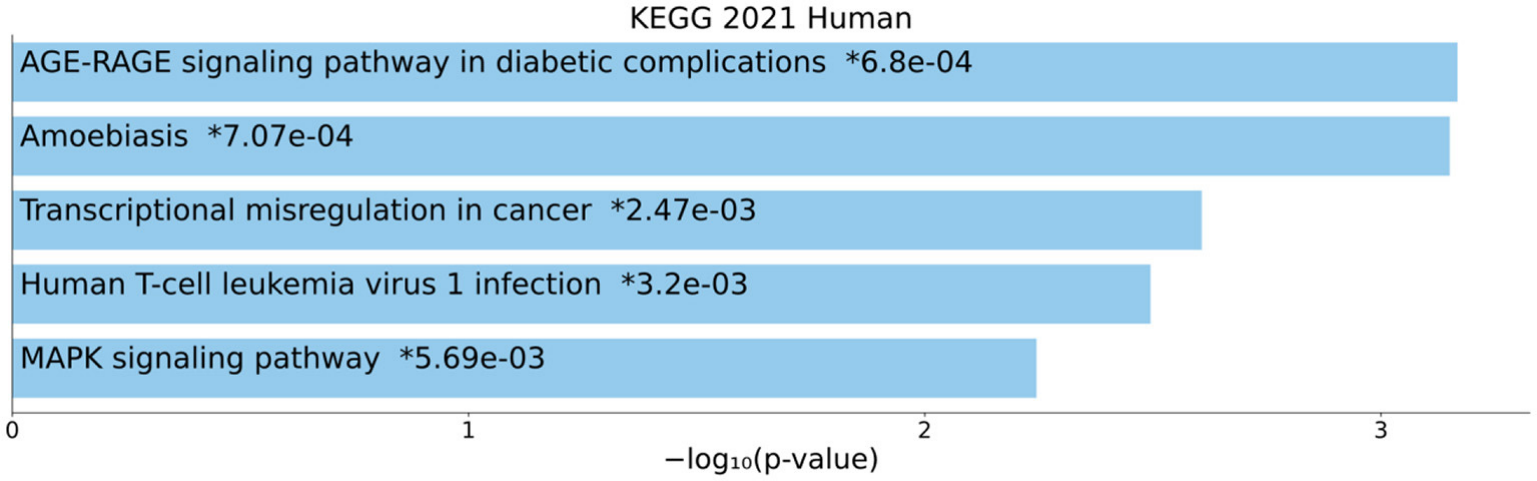
Kyoto Encyclopedia of Genes and Genomes (KEGG) pathway enrichment analysis. The top five enriched pathways are displayed along with the corresponding *P*-values in a bar graph. Those with significant *P*-values (i.e., <0.05) are matched with the colored bars. A *P*-value (shown with *) shows the significant adjusted *P*-value (< 0.05).

The KEGG pathway enrichment analysis was performed on all DEmRNAs in the ceRNA network. Hence, the top enriched KEGG pathways are mentioned as follows: “AGE-RAGE signaling pathway in diabetic complications”, “Amoebiasis”, “Transcriptional misregulation in cancer”, “Human T-cell leukemia virus 1 infection”, and “MAPK signaling pathway” (Figure 5).

## Discussion

Several studies have shown brain BA9, and BA10 regions are affected by SCZ pathology and cortical dysfunction. The involvement of these regions is associated with cognitive impairment (7, 8, 30, 31). Furthermore, it has been suggested that ceRNA regulatory axes and their related networks contribute to a variety of neurological disorders (32). Organ, cell, and the subcellular environment have important impacts on the expression level of the ceRNA. Different RNA transcripts such as lncRNAs, circular RNAs (circRNAs), pseudogenes, and mRNAs can be involved in the ceRNA network. LncRNAs are one of the main elements of the ceRNA network and have a pivotal role in both normal and pathological conditions (33). It is believed that lncRNAs have different expression patterns depending on the development stage, cell type, and organ. The expression of lncRNAs is strictly regulated based on their tissue specificity and subcellular localization (34). The lncRNA-associated ceRNA axes may have a critical role in SCZ pathogenesis, according to the aforementioned theoretical assumptions. In the current study, we discovered the DElncRNA-miRNA-DEmRNA, which included two key lncRNAs, seven key miRNAs, and eight key mRNAs.

We found two major lncRNAs (*PEG3-AS1, MIR570HG*) in this investigation. It has been demonstrated that *PEG3-AS1* has been correlated with acute myeloid leukemia (35), colorectal cancer (36), and head and neck squamous cell carcinoma (37); however, the exact function and mechanisms of *PEG3-AS1* are poorly understood. As far as we know, the correlation between *PEG3-AS1* and SCZ has been reported in this study for the first time; thus, additional research is required to confirm our findings. *MIR570HG* (originally designated as *LINC00969*) is another new lncRNA that has been reported to be linked to intervertebral disk degeneration (IDD) (38). Zhao et al. (38) studied the differential expression of lncRNAs in patients with IDD in comparison to control subjects affected with spinal cord injury using RNA-sequencing combined with quantitative real-time PCR and identified several differentially expressed lncRNAs in IDD patients. Among them, *LINC00969* lncRNA was among the top 10 upregulated lncRNA in nucleus pulposus samples of IDD patients. To our knowledge, our study is the first to identify a link between *MIR570HG* and SCZ, so additional research should be done to verify the reported findings. In line with our result, a previous study showed that *LINC00969* positively regulates the expression of the thioredoxin-interacting protein (*TXNIP*) and increases IDD degeneration by acting as a ceRNA for *miR-335-3p* and sponging it and modulating activation of NLRP3 inflammasome (39). Moreover, Lee et al. (40) explored possible gene-gene interaction in susceptibility to SCZ by intensively searching for SNP-SNP interactions in three GWAS datasets. They observed that one of these SNP-SNP interactions could be understood as the interaction between *FHIT* and *LINC00969* (40). *FHIT* and *LINC00969* were discovered to have an expression in the brain and could represent a novel discovery in SCZ research (40).

We predicted that the sponging of seven key miRNAs (*hsa-miR-124-3p, hsa-miR-17-5p, hsa-miR-181a-5p, hsa-miR-191-5p, hsa-miR-26a-5p, hsa-miR-29a-3p, hsa-miR-29b-3p*) by key lncRNAs might influence target genes. By binding to the non-transcript region of the target gene, miRNAs can regulate the expression of the gene. This might have an impact on cellular signaling and biological pathways, which may influence the initiation and course of SCZ (41). Among the key miRNAs, the correlation between *hsa-miR-124-3p, hsa-miR-17-5p*, hsa-miR-29a-3p, and *hsa-miR-29b-3p* and SCZ has been studied, already. In accordance with the multi-hit and multi-layered genetic mechanisms implicated in this and other forms of SCZ, Ying et al. (42) proposed a potential role for *miR-17-5p* and *hsa-miR-124-3p* perturbation involving genes affected by rare genome-wide copy number variations in the elevated risk for SCZ in 22q11.2 deletion syndrome. In accord with our results, elevated levels of *miR-124-3p* in SCZ patients compared with healthy controls was reported in a previous investigation (43). In another study, Xu et al. (44) reported upregulation of *miR-124-3p* in patients with SCZ. They also identified a composite feed-forward loop consisting of EGR1-miR-124-3p-SKIL that may have clinical significance (44). In a previous research, it has been shown that expression of *miR-29a-3p* decreased in the prefrontal cortex of SCZ patients compared to individuals without psychiatric disorders (45), which is in line with our findings. Finally, it has been indicated that *miR-29b-3p* is one of the SCZ susceptibility loci (46). Although these findings support our results, additional functional studies must be done to validate our anticipated ceRNA axes.

In this study, we performed KEGG enrichment analysis on DEmRNAs and found that significantly enriched pathways include: “AGE-RAGE signaling pathway in diabetic complications”, “Amoebiasis”, “Transcriptional misregulation in cancer”, “Human T-cell leukemia virus 1 infection”, and “MAPK signaling pathway”. Advanced glycation end products (AGEs), produced through glycation of lipids or proteins with reducing sugars in a non-enzymatic manner, have been associated with different illnesses, like cardiovascular complications in patients with diabetes mellitus (47), chronic renal failure (48), and Alzheimer's disease (49). Plasma pentosidine AGEs were shown to be connected to SCZ in earlier investigations (50–52) and serve as a helpful biomarker for the treatment-resistant-like phenotype (53). Elevated levels of AGEs may result in psychotic symptoms by inducing brain inflammation (54). In fact, AGEs cause elevation of proinflammatory cytokines through binding to a membrane-bound receptor, RAGE, that is present on membrane of astrocytes, microglia, and neurons (55). The blood and cerebrospinal fluid of SCZ patients have elevated levels of pro-inflammatory indicators, such as cytokines (56). A comprehensive epidemiological investigation has conclusively shown that severe infections and autoimmune illnesses are risk factors for SCZ (57). The vulnerability-stress-inflammation paradigm may be useful for elucidating the role of inflammation in SCZ since stress can enhance pro-inflammatory cytokines and even lead to a persistent pro-inflammatory state (56). SCZ is characterized by risk genes that induce inflammation, environmental stress factors, and immune system modifications. Typical abnormalities in dopaminergic, serotonergic, noradrenergic, and glutamatergic neurotransmission observed in SCZ have also been seen in low-level neuroinflammation, and hence may be important contributors to the onset of SCZ symptoms (56). Neuroimaging evidence of volume loss in the central nervous system (CNS) and microglial activation provides additional support for the importance of a low-level neuroinflammatory process in SCZ (56). The early twentieth century-famous infectious theory of psychosis (58) has now gained additional scientific justification (59–62). It has been shown that SCZ is a multifactorial disease that results from the interaction of SCZ susceptibility genes with environmental factors. In addition to lots of SCZ susceptibility genes, viral infections during the prenatal or adult period and toxoplasmosis or Lyme disease also can have a role in this disease (63). The discovery of infectious pathogens that are linked to SCZ etiopathogenesis might result in the development of novel approaches to prophylaxis, diagnosis, and therapy of SCZ (58). The gene expression patterns playing an important role in creating and sustaining particular cell states are exactly regulated by a substantial number of transcription factors, cofactors, and chromatin regulators. Perturbation of these gene expression patterns can contribute to a wide range of illnesses, from cancers to neurological disorders like SCZ (64). According to available data, SCZ may be caused by changes to the way that different neurotransmitter systems signals are integrated. Recently, dysregulation of cyclic adenosine monophosphate (cAMP) and mitogen-activated protein kinase (MAPK) signaling pathways has been observed in SCZ patients in brain dorsolateral prefrontal cortex and anterior cingulate (65).

Primarily we found eight mRNAs in the ceRNA network, including *EGR1, ETV1, DUSP6, PLOD2, CD93, SERPINB9, ANGPTL4*, and *TGFB2*. Among these key mRNAs, *EGR1, DUSP6, PLOD2, CD93, SERPINB9*, and *TGFB2* have already been mentioned to be related to the SCZ. The *EGR1* gene is from the *EGR* family of Cys2-His2-type zinc-finger proteins, which encodes the early protein (66). Additionally, it is involved in cell proliferation, female reproduction, immune response, cell growth, neuronal plasticity, and memory formation (67). EGR1 is a nuclear protein functioning in neural development (as a transcriptional regulator) (67). Animal studies revealed that brain function, cognitive aging, and antipsychotic drug administration could affect the *EGR1* gene (68, 69). Moreover, downregulation of *EGR1* has been reported in several studies in SCZ patients (44, 70–73). For instance, an analysis of reverse transcription-quantitative PCR illustrated a considerable decline in the *EGR1* expression level in peripheral blood mononuclear cells and the prefrontal cortex in SCZ patients (compared with controls) (44, 70, 71, 73). Also, compared to the control samples, the downregulation of *EGR1* in the anterior cingulate cortex of SCZ samples was observed by Ramaker et al. (72). These findings are similar to our results which declare that the *EGR1* gene is related to the pathophysiology of SCZ. Nevertheless, some other expression studies showed up-regulation of the *EGR1* gene in fibroblasts, peripheral blood cells (74), and in post-mortem superior temporal cortex of SCZ (75), in which these incompatible observations could be justified by SCZ's heterogeneity, medications, and epigenetic processes (76). The human *DUSP6* gene is located at 12q22–q23 (77), overlapping a locus correlated with susceptibility to bipolar disorder. This gene's genetic association with SCZ and bipolar disorder has been represented (78). Furthermore, *DUSP6* is referred to as MAP Kinase Phosphatase 3 (*MKP3*) (a cytoplasmic phosphatase), which is more selective for extracellular signal-regulated kinase1/2 (*ERK1/2*) compared to other MAPK isoforms (79, 80). Additionally, PLOD2 is recognized as an enzyme for mediating the formation of stabilized collagen cross-links in collagen (through the hydroxylation of lysyl residues) (81). Our results from the up-regulation of *PLOD2* are concordant with previous studies (82). A negative regulator in astrogenesis and participating in the regulation of CNS inflammation is *CD93*, familiar for its immune functions (83, 84). CD93 was widely and ubiquitously expressed in the brain and may be involved in the regulation of innate and adaptive immunity in the CNS. Similarly, we reported results from the bioinformatics analysis, such as up-regulation of *CD93* in ceRNA axes in the hippocampus, BA46, and striatum samples obtained from patients with SCZ (14). An intracellular inhibitor of the cytotoxic protease granzyme B (grB) is *SERPINB9* which was previously named *PI-9* in humans (*SPI6* in mice) (85). It is generated in CD8+ T and NK cells (with a nucleo-cytoplasmic distribution), which is vital for these cells' protection against grB-mediated apoptosis (85). With regards to the former investigations, SERPINB9 protein was upregulated in SCZ organoids (86), which meets our results. The TGFBs manage various cells' growth, differentiation, and function and have been involved in several disease processes (87). Notably, according to the reports, TGFB2 seems to be a critical driver in the dysregulation of numerous genes related to SCZ (88). Moreover, aberrant epigenetic regulation of *TGFB2* and alteration in TGFβ signaling might contribute to loss or reversal of brain laterality in SCZ cases. A previous study revealed that clozapine exposure is correlated with down-regulation of *ANGPTl4* (89). Clozapine, an uncommon antipsychotic treatment, is used for SCZ patients with other antipsychotic drug resistance (90). *ANGPTL4* is linked with triglyceride and high-density lipoprotein cholesterol levels (91); hence it could be related to the higher risk of type 2 diabetes in SCZ patients treated with clozapine drug (89, 92). Delplanque et al. reported that *ETV1*, another key mRNA in the ceRNA network, correlates with spinocerebellar ataxia 21 (another mental disorder) (93). A member of the ETS family (E twenty-six) of transcription factors is encoded by *ETV1*. This transcription factor can attach to distinct DNA sequences in the promoter/enhancer regions of genes and regulate several biological pathways (94).

It should be noted that a number of technical factors, including various methodologies, patient characteristics, sample preparation, data analysis, and platforms, could have an impact on the gene expression profiles. Furthermore, small sample size may compromise statistical power. On the other hand, potential covariates (e.g., RNA extraction quality, post-mortem interval, and antipsychotic treatment) were not statistically controlled. Finally, our findings must be supported by confirmatory experimental work and comparisons to reanalysis of modified microarray gene expression.

## Conclusion

Our study discovered ceRNA axes associated with lncRNAs, which may be important for SCZ. These axes consist of eight key mRNAs (*EGR1, ETV1, DUSP6, PLOD2, CD93, SERPINB9, ANGPTL4, TGFB2*), two key lncRNAs (*PEG3-AS1, MIR570HG*), and seven key miRNAs (*hsa-miR-124-3p, hsa-miR-17-5p, hsa-miR-181a-5p, hsa-miR-191-5p, hsa-miR-26a-5p, hsa-miR-29a-3p, hsa-miR-29b-3p*). Despite the need to understand the functions of these axes, this work provides possible research targets for looking at molecular pathways that might be important for SCZ pathogenesis.

## Data availability statement

Publicly available datasets were analyzed in this study. This data can be found here: https://www.ncbi.nlm.nih.gov/geo/query/acc.cgi?acc=GSE17612.

## Author contributions

HS, MR, MT, and BMH wrote the draft and revised the manuscript. MT, SMN, and SB designed and supervised the study. MG, MRA, SA, and MS-B collected the data and performed the bioinformatic analysis. All authors read and approved the submitted version.

## Conflict of interest

The authors declare that the research was conducted in the absence of any commercial or financial relationships that could be construed as a potential conflict of interest.

## Publisher's note

All claims expressed in this article are solely those of the authors and do not necessarily represent those of their affiliated organizations, or those of the publisher, the editors and the reviewers. Any product that may be evaluated in this article, or claim that may be made by its manufacturer, is not guaranteed or endorsed by the publisher.
