# Supplementary material for: Identification of key long non-coding RNA-associated competing endogenous RNA axes in Brodmann Area 10 brain region of schizophrenia patients
Source: Front Psychiatry. 2022 Nov 3;13:1010977. doi: 10.3389/fpsyt.2022.1010977 (PMC9671706; doi:10.3389/fpsyt.2022.1010977)
Supplement: Supplementary file 3 [file Data_Sheet_1.docx]

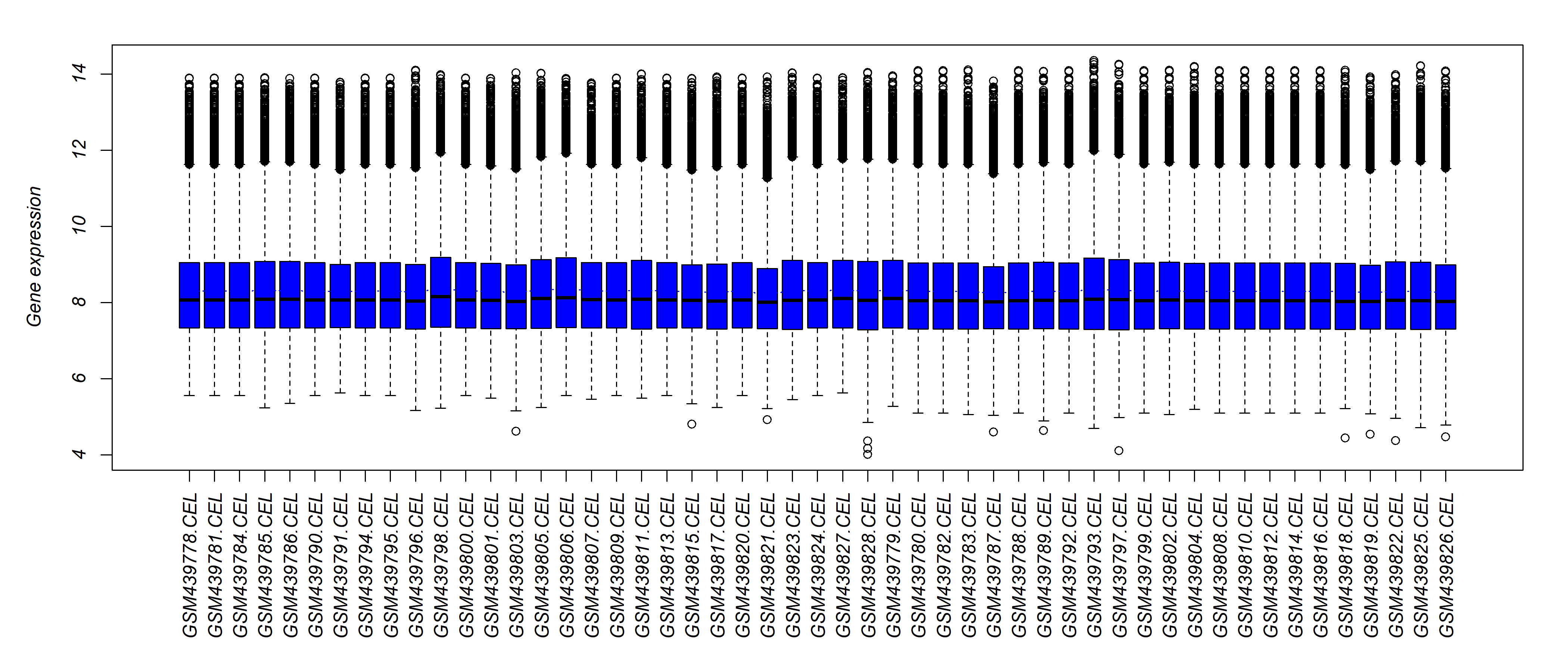


**Figure S1.** Boxplot for GSE17612 dataset. The horizontal axis displays the sample names, while the vertical axis displays the gene expression.
